# Supplementary material for: Genomic Organization of the B3-Domain Transcription Factor Family in Grapevine (Vitis vinifera L.) and Expression during Seed Development in Seedless and Seeded Cultivars
Source: Int J Mol Sci. 2019 Sep 14;20(18):4553. doi: 10.3390/ijms20184553 (PMC6770561; doi:10.3390/ijms20184553)
Supplement: Supplementary file 1 [file ijms-20-04553-s001.zip › Supplementary data/Supplementary Table 1-5.docx]

**Table S1:** Tandem duplication events in grapes B3 genes

| Cluster number | Chromosome | Gene | Start Site | End Site |
| --- | --- | --- | --- | --- |
| 1 | 3 | VvREM1 | 4170184 | 4173696 |
|  | 3 | VvREM2 | 4176729 | 4178437 |
|  | 3 | VvREM3 | 4180639 | 4183390 |
| 2 | 3 | VvREM4 | 4859928 | 4864681 |
|  | 3 | VvREM5 | 4874233 | 4875367 |
|  | 3 | VvREM6 | 4879760 | 4881735 |
|  | 3 | VvREM7 | 4886639 | 4887736 |
|  | 3 | VvREM8 | 4887975 | 4921315 |
| 3 | 7 | VvREM10 | 14390670 | 14392515 |
|  | 7 | VvREM11 | 14403658 | 14404557 |
| 4 | 18 | VvREM18 | 9665889 | 9667070 |
|  | 18 | VvREM19 | 9669874 | 9677462 |

**Table S2:** Synteny blocks of B3 genes within grape genomes.

| Region 1 (Grape) Region 2 (Grape) Gene in the synteny region | | | | | | | | |
| --- | --- | --- | --- | --- | --- | --- | --- | --- |
| ID① | Chr | Start | Stop | Chr | Start | Stop | Gene 1 | Gene 2 |
| 19 | 12 | 21751538 | 22690315 | 18 | 27975112 | 29342811 | VvARF13 | VvARF17 |
| 105 | 6 | 7674586 | 8985737 | 8 | 8767790 | 10972180 | VvVAL1 | VvVAL2 |
| 115 | 7 | 16292195 | 17030189 | 3 | 3383813 | 4306720 | VvREM12 | VvREM1 |
| 104 | 6 | 2948720 | 7369025 | 8 | 12706428 | 16328346 | VvARF4 | VvARF7 |
| 28 | 6 | 2948720 | 7369025 | 8 | 12706428 | 16328346 | VvARF5 | VvARF7 |
| 69 | 18 | 8368864 | 9355161 | 7 | 16300878 | 17030189 | VvREM17 | VvREM12 |
| 81 | 2 | 236398 | 2725052 | 15 | 16064252 | 19083025 | VvARF2 | VvARF14 |
| 59 | 18 | 8406079 | 9498340 | 3 | 3554244 | 4423196 | VvREM17 | VvREM1 |
| 60 | 18 | 9669874 | 10780468 | 3 | 4866536 | 5897060 | VvREM19 | VvREM5 |
| 81 | 2 | 2549060 | 2661460 | 15 | 15539622 | 6742157 | VvRAV3 | VvRAV7 |
| 2 | 1 | 5375293 | 2241578 | 14 | 22390052 | 13579146 | VvRAV1 | VvRAV6 |

**Table S3:** Synteny blocks of B3 genes between grape and *Arabidopsis*

| Region 1 (Arabidopsis) | | | | Region 2 (Grape) | | | Gene in the synteny region | |
| --- | --- | --- | --- | --- | --- | --- | --- | --- |
| ID① | Chr | Start | Stop | Chr | Start | Stop | Gene 1 | Gene 2 |
| 123 | 1 | 18234724 | 18405563 | 18 | 3823723 | 5670765 | AT1G49475 | VvREM6 |
| 120 | 1 | 31170 | 54692 | 8 | 2335462 | 2537782 | AT1G01030 | VvRAV3 |
| 607 | 5 | 23558114 | 23906977 | 3 | 6110123 | 9331555 | AT5G58280 | VvREM9 |
| 228 | 2 | 12043482 | 13005600 | 8 | 3267313 | 9016608 | AT2G28350 | VvARF4 |
| 631 | 5 | 1856008 | 1935644 | 8 | 16424080 | 17043399 | AT5G06250 | VvRAV4 |
| 252 | 2 | 12852372 | 12985043 | 7 | 9230232 | 10767781 | AT2G30470 | VvVAL2 |
| 58 | 3 | 22671002 | 22915325 | 15 | 16989617 | 19781736 | AT1G01030 | VvRAV7 |
| 465 | 4 | 16428480 | 16447773 | 3 | 4887975 | 5311431 | AT4G34400 | VvREM8 |
| 562 | 5 | 17073779 | 17341569 | 3 | 7580653 | 9695345 | AT5G42700 | VvREM17 |
| 328 | 3 | 6484068 | 6716099 | 15 | 3794273 | 5670765 | AT3G18960 | VvREM6 |
| 285 | 3 | 9809950 | 11269228 | 14 | 24706500 | 30137019 | AT3G26790 | VvABI3-3 |
| 328 | 3 | 6611198 | 6783166 | 18 | 7901913 | 9407321 | AT3G19184 | VvREM1 |
| 359 | 3 | 8997370 | 9085048 | 7 | 9431080 | 12072090 | AT3G24650 | VvABI3-1 |
| 228 | 2 | 12852372 | 12985043 | 6 | 8748759 | 8957227 | AT2G30470 | VvVAL1 |
| 318 | 3 | 651119 | 6883166 | 18 | 9025103 | 9126342 | AT3G19184 | VvREM17 |
| 261 | 3 | 9494302 | 9333719 | 1 | 3333709 | 2088886 | AT3G25730 | VvRAV1 |

**① ID** means the synteny block ID in the synteny analysis (Nucleic Acids Res, 2012. 40(7):e49.)

**Table S4**: Segmental duplication pairs of B3 genes between grape and tomato

| **Region 1 (Tomato)** | | **Region 2 (Grapes)** | |
| --- | --- | --- | --- |
| **Chromosome** | **Gene** | **Chromosome** | **Gene** |
| 6 | Solyc06g075150.2 | 6 | ARF4 |
| 11 | Solyc11g069500.1 | 8 | ARF7 |
| 8 | Solyc08g013690.1 | 2 | RAV3 |
| 10 | Solyc10g083210.1 | 8 | RAV4 |
| 8 | Solyc08g013690.1 | 15 | RAV7 |
| 6 | Solyc06g083590.2 | 7 | ABI3-1 |
| 2 | Solyc02g094460.1 | 14 | ABI3-3 |
| 10 | Solyc10g075030.1 | 6 | VAL1 |
| 6 | Solyc06g082520.2 | 8 | VAL2 |
| 2 | Solyc02g079020.2 | 19 | VAL3 |
| 2 | Solyc02g079020.2 | Un | VAL4 |
| 4 | Solyc04g079130.2 | 3 | REM1 |
| 2 | Solyc02g065340.1 | 3 | REM5 |
| 6 | Solyc06g007530.2 | 6 | REM9 |
| 4 | Solyc04g079130.2 | 7 | REM12 |
| 1 | Solyc01g081400.2 | 15 | REM15 |
| 3 | Solyc03g111500.2 | 17 | REM16 |
| 2 | Solyc02g090710.2 | 18 | REM17 |
| 2 | Solyc02g090170.2 | 18 | REM19 |

**Table S5:** Primer sequences used in expression analysis of B3 genes in grape

| **Gene** | **Forward and reverse primer sequence (5'~3')** | |
| --- | --- | --- |
| VvRAV3 | F | CGGGAGACATTGTGAGTTT  CACTGCTATTTACGGGTACT |
|  | R |  |
| VvRAV4 | F | CGCTGACCGCTTCTTTATT  GGTTCGGACTCATCCATTTG |
|  | R |  |
| VvRAV7 | F | CGGTGTTGGAGAATCAGGTAA  GGACGGACTGGGAGAATGGTA |
|  | R |  |
| VvABI3-1 | F | CAGAAGAAGCGAATCAGCG GGTGTAAGGAGAAGGCAAAG |
|  | R |  |
| VvABI3-2 | F | AGCCTGGTGATTTGTTTGTAGT  CTGTATTGAGGTCCCTTGTTCT |
|  | R |  |
| VvABI3-3 | F | ATCATCCGCCATCAACCT  ATGAGAATCCCTTCCTTTG |
|  | R |  |
| VvVAL1 | F | TGGAAGAAGGGATGGACTA   ACGCTTGTGGCATAAACTG |
|  | R |  |
| VvVAL2 | F | GCCCAATAATAACAGCAGGAT GAAGAATGCCAGAGTAACCA |
|  | R |  |
| VvVAL3 | F | GCAATGCTAACAGCCAGAT  TGTCCCTTCGGTTCTATC |
|  | R |  |
| VvVAL4 | F | GATTAGAACCAGAAGGGAAGC  ACCCACCATTGCCAGTCTT |
|  | R |  |
| VvREM1 | F | AGTGCCCATTGAGCCTTTG AGACCCAGCCAGAACCCA |
|  | R |  |
| VvREM2 | F | TTCCAAACACCTCCAGACCA  GCTCGTAAGAAGCCGACATT |
|  | R |  |
| VvREM3 | F | TTCTGAGTTACCAAGCCTGAC  GACCCTCACAATGAACACCTT |
|  | R |  |
| VvREM5 | F | TGAGGATGGGTGGAAGGAG   CAGGGAGCCAACTGGGTAT |
|  | R |  |
| VvREM6 | F | TTGGGTTCTGCTTCTCCTG ATTGAATGCCTATGTCTCGG |
|  | R |  |
| VvREM8 | F | ATCTTCTAACAAGGGAGTGGT  CCTCCTTCCTGGTCGTATT |
|  | R |  |
| VvREM9 | F | GCGAGGACCATCTACCAA  TCTCCAACCACCACTAAG |
|  | R |  |
| VvREM10 | F | GGCGATGGTGAGGTTTGGTT TTGTTGTCTGCCTGGCTGGA |
|  | R |  |
| VvREM11 | F | GGACAATGACTCCGACAG  CCTTGACTAAACCGCACAC |
|  | R |  |
| VvREM12 | F | TATCGCAGACAAGACGGGACT TTGGCTCAACTAAATGGAAGACC |
|  | R |  |
| VvREM14 | F | GACAGACTGCTCATCCCAACA  CTGCCAACCGTTCAGAAAGTA |
|  | R |  |
| VvREM15 | F | AACACTCCCAGAAACCCCACATA  GCAGGAATCACAGCAGAAGGAA |
|  | R |  |
| VvREM16 | F | CAGCCAACCTGTCCTGTATT   TATGCCCTTGAGTTCGTT |
|  | R |  |
| VvREM17 | F | AACAAGGCTTACCAGGCTCAA  TCGGATACTCATCACCCTCTT |
|  | R |  |
| VvREM19 | F | CTTTCGTCCGTGGTAATG  ACACCCTGGAGTAAACCC |
|  | R |  |
| Actin1 | F | GATTCTGGTGATGGTGTGAGT GACAATTTCCCGTTCAGCAGT |
|  | R |  |
| EF1-α | F | AGGAGGCAGCCAACTTCACC   CAAACCCTGCATCACCATTC |
|  | R |  |
