# Supplementary figures and images for: Genomic Organization of the B3-Domain Transcription Factor Family in Grapevine (Vitis vinifera L.) and Expression during Seed Development in Seedless and Seeded Cultivars
Source: Int J Mol Sci. 2019 Sep 14;20(18):4553. doi: 10.3390/ijms20184553 (PMC6770561; doi:10.3390/ijms20184553)

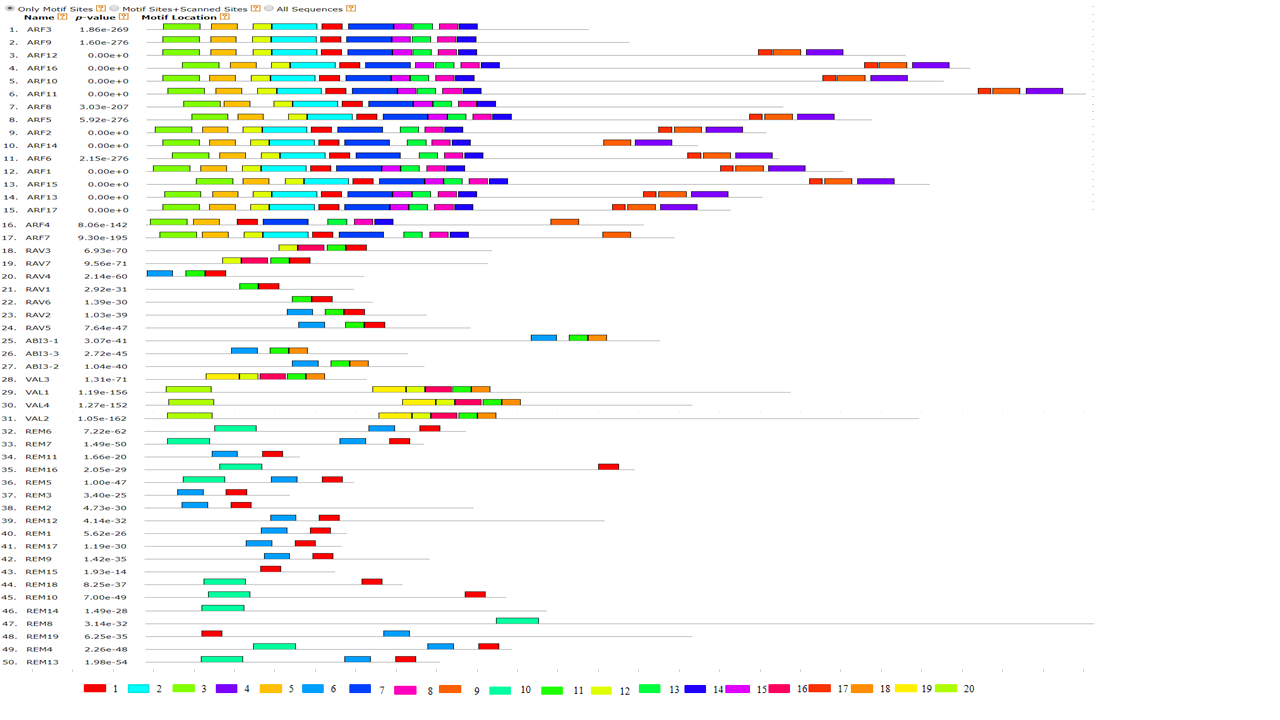

Supplement: Supplementary file 1 [file ijms-20-04553-s001.zip › Supplementary data/Supplementary Fig. 1.tif]
